# The Physical Improvement League

**Published:** 1905-07-08

**Authors:** 


The Hospital.

A Journal of

The Medical Sciences and Hospital Administration.

Vol. XXXVIII.—No. 980. SATURDAY, JULY 8, 1905.

### THE PHYSICAL IMPROVEMENT LEAGUE.

The great meeting at the Mansion House for the foundation of a League of Physical Improvement, to the approach of which we called attention last week, must, we think, have been entirely satisfactory to its promoters. The attendance was very large, and the speakers — more especially Mrs. Bramwell Booth—were received with a degree of favour which might almost be described as enthusiasm. The only discordant note was struck by a lady with a singularly ear-piercing voice, who, after the second resolution had been seconded, inquired in shrill tones whether an amendment had been handed in, and, if so, whether it had been rejected as out of order. The Lord Mayor replied that no amendment had been received on which the inquirer sat down. The Bishop of Ripon, as chairman of the Executive Council, declared the objects of the projected League to be not only the creation of increased facilities for leading healthful lives, but also the focussing and combining of a great deal of scattered effort which is now being made in the same directions by individuals or organisations often ignorant of the kindred operations of others—a condition which serves materially to curtail the usefulness of their efforts. The Bishop declared that the League did not wish to supersede other workers, but only to render assistance in the direction of uniting their efforts into one organised whole.

The Lord Chief Justice, in an eminently manly and telling speech, referred to his opportunities at the bar and on the bench of studying the causes of criminality; and among these he placed, as second indeed to drink, but to drink only, the fact that a large proportion of the children of the poorer classes have no other playground than the streets—places in which their eyes and ears are early familiarised with the conduct and with the language of the criminal classes. Mr. Alderman and Sheriff Strong dwelt upon the increasing competition of commerce, and upon the national need for strong muscles and capable brains in order that this competition may be successfully encountered; and Sir James Crichton Browne, enlarged convincingly upon his favourite topic of the importance of physical exercise as a means of developing the motor and other centres of the brain, and of the necessity of supplying conditions essential to their welfare.

All this was without doubt excellent, but nevertheless, as we have indicated above, the honours of the day were with Mrs. Bramwell Booth, whose admirable elocution rendered every word that she uttered distinctly audible to the whole of the assembly, and whose speech lifted the discussion to a higher plane than any other speaker had touched. Her argument was mainly an eloquent plea for the better recognition of the dignity, and for a better discharge of the duties, of maternity; and she intimated, not obscurely, that the physical degeneracy of the people, in so far as it exists, has a moral side which is perhaps more important than the material and more urgently requires attention and effort. She repudiated, with something like scorn, the idea that parents should be "relieved" of the duty of caring for their offspring, or even of the duty of feeding them; and quoted a Spanish proverb to the effect that an ounce of mother is worth a ton of priest. It was impossible not to feel that she had touched the heart of the problem, and that the absence of sound principles of conduct is at the root of many of the evils which the meeting had assembled to deplore, and, so far as possible, to remedy. To what extent the desired rectification will be effected by the means indicated by the majority of the speakers is, we think, somewhat doubtful. The consequences of conduct, in the largest sense of the word, are as penetrating as they are far-reaching; and the roots of conduct are often deeply laid in the habits of earlier generations.

We have to turn to the teachings of history before we can fully comprehend the different standpoints from which two very important matters—the affection and respect due to mothers, and the folly and sinfulness of waste—are regarded in England on the one hand, and in France on the other. Mrs. Bramwell Booth's appeal, which, we greatly fear, would have passed altogether over the heads of a working-class audience in this country, would have profoundly touched a French one. With regard to waste, which lies at the foundation of nearly all poverty, the working classes of this country have exalted it into a principle of action. A French *bonne* is careful alike of her employer's goods and of her own, mainly, perhaps, because waste of any sort is hateful to her, and is opposed to the principles in which she has been brought up, and to which she has been accustomed all her life. An English servant, as a rule, wastes as much of her employer's substance as possible, and thinks her own dignity enhanced by the extent to which she can increase the coal and butcher's bills. When she marries and has children she continues to be wasteful in her own home, not from any lack of self-interest, but partly from inveterate habit and partly from invincible ignorance. Her children are badly fed, not from poverty, but because she has never learnt how to make the most of her resources. Such physical degeneration as may exist is largely due to her inefficiency; and her increasing inefficiency is itself mainly attributable to the political and sectarian influences which, during the last half-century, have selected as their battle-ground the field of national education.

